# Rounded leaf end effect of multileaf collimator on penumbra width and radiation field offset: an analytical and numerical study

**DOI:** 10.1515/raon-2015-0023

**Published:** 2015-08-21

**Authors:** Dong Zhou, Hui Zhang, Peiqing Ye

**Affiliations:** Department of Mechanical Engineering, Tsinghua University, Beijing, China

**Keywords:** multileaf collimator, rounded leaf end effect, penumbra width, radiation field offset, Monte Carlo simulation

## Abstract

**Background:**

Penumbra characteristics play a significant role in dose delivery accuracy for radiation therapy. For treatment planning, penumbra width and radiation field offset strongly influence target dose conformity and organ at risk sparing.

**Methods:**

In this study, we present an analytical and numerical approach for evaluation of the rounded leaf end effect on penumbra characteristics. Based on the rule of half-value layer, algorithms for leaf position calculation and radiation field offset correction were developed, which were advantageous particularly in dealing with large radius leaf end. Computer simulation was performed based on the Monte Carlo codes of EGSnrc/BEAMnrc, with groups of leaf end radii and source sizes. Data processing technique of curve fitting was employed for deriving penumbra width and radiation field offset.

**Results:**

Results showed that penumbra width increased with source size. Penumbra width curves for large radius leaf end were U-shaped. This observation was probably related to the fact that radiation beams penetrated through the proximal and distal leaf sides. In contrast, source size had negligible impact on radiation field offset. Radiation field offsets were found to be constant both for analytical method and numerical simulation. However, the overall resulting values of radiation field offset obtained by analytical method were slightly smaller compared with Monte Carlo simulation.

**Conclusions:**

The method we proposed could provide insight into the investigation of rounded leaf end effects on penumbra characteristics. Penumbra width and radiation field offset calibration should be carefully performed to commission multileaf collimator for intensity modulated radiotherapy.

## Introduction

Multileaf collimator system was introduced as a replacement of shielding block for beam shaping and beam intensity modulation, which has become an essential component for modern radiation therapy and a standard of care for radiation oncology facilities.^1^ Penumbra characteristics of multileaf collimator are closely related to healthy tissues involvement, which is of interest to medical physicists, dosimetrists and radiation oncologists.^2^

Single-focused multileaf collimator is characterized by linear leaf motion perpendicular to collimator rotation axis, which has been widely used by virtue of its compact space and simplified structures. The rounded leaf end design of single-focused multileaf collimator for following beam divergence has a strong impact on penumbra characteristics.^3^ In order to avoid tumour underdose and normal tissue overdose, the rounded leaf end effect of single-focused multileaf collimator on penumbra characteristics should be carefully modelled in treatment planning system, otherwise it would result in dose error particularly when sharp dose gradient is intended for stereotactic body radiotherapy.

For the purpose of precision radiation therapy, intensive research efforts have been made on the dosimetric measurement and Monte Carlo simulation of multileaf collimator systems.^4^,^5^ Studies have revealed that dosimetric characteristics of multileaf collimator are influenced by the factors, including but not limited to geometry of treatment modality, radiation source properties, leaf end shape and leaf position with respect to central axis.^6^ It was found that dosimetric penumbra of multileaf collimator is the combined effect of geometric penumbra, transmission penumbra and phantom scatter.^7^ Quality assurance has been implemented to determine penumbra width and the offset between light field edge and radiation field edge during commissioning of multileaf collimator.^8^,^9^ Results have shown that penumbra width and radiation offset are leaf position dependent and largely attributed to leaf end shape. It is reported that the projected leaf position on scoring plane, light field edge and radiation field edge follow a nonlinear relationship. Calibrations of leaf position offset and radiation field offset were performed to minimize the error between planned doses and delivered doses.^10^,^11^ Rule of half-value layer^12^ has been proposed for calculation of radiation field offset based on geometrical approach.^13^ However, previous studies were confined to single source energy distribution, normally simplified as Gaussian shaped, and limited leaf ends in the shape of circular arc were investigated. There is a lack of consistency in the quantitative study into rounded leaf end effect on penumbra characteristics of multileaf collimator in literature. Besides, there is no literature available, to our knowledge, reporting on algorithms of leaf position calculation and radiation field offset correction for large radius leaf end.

Consequently, the aim of this study was to explore the rounded leaf end effect and efforts were made to reveal the source energy distribution and leaf end shape related penumbra characteristics. An analytical method for radiation field offset correction was developed and numerical simulation with various leaf end radii and source sizes was conducted based on Monte Carlo codes.

## Materials and methods

In this section, leaf positions are classified and geometry based algorithms for radiation field offset correction are developed. With treatment head modelling, Monte Carlo simulation is introduced to investigate the rounded leaf effect on penumbra characteristics. Data processing techniques for deriving penumbra width and radiation field offset are proposed.

### Algorithms for leaf position calculation and radiation field offset correction

Leaf positions on scoring plane are divided into projected leaf end position (nominal leaf position), light field edge (geometric leaf position), and radiation field edge (physical leaf position).^14^ Nominal leaf position is usually calibrated so that it corresponds to the light field edge or the radiation field edge. In this study, nominal leaf position is designated to coincide with the projected leaf position without calibration.

**FIGURE 1.**
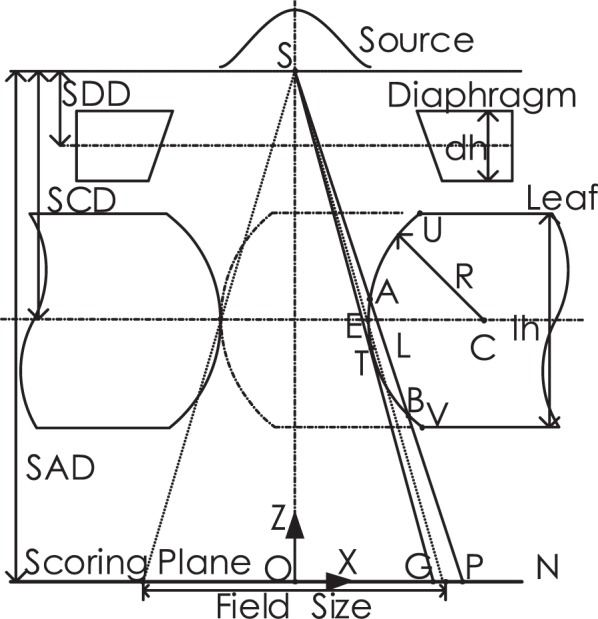
Components of treatment head are comprised of source, diaphragm, multileaf collimator, and scoring plane. Leaf positions on scoring plane are classified into nominal leaf position N, geometric leaf position G and physical leaf position P. SAD = source to axis distance; SCD = source to collimator distance; SDD = source to diaphragm distance

As depicted in Figure 1, mechanical leaf position is referred to as the leaf tip location relative to collimator rotation axis, which is shown as point E. Nominal leaf position, geometric leaf position and physical leaf position on the scoring plane are represented by point N, point G, and point P, respectively. Leaf position offset (LPO) is defined as the distance between geometric leaf position and nominal leaf position. Radiation field offset (RFO) is defined as the distance between physical leaf position and geometric leaf position. The term of physical-nominal offset (PNO) is proposed, which is defined as the distance between physical leaf position and nominal leaf position.

Place the origin of coordinate in coincidence with isocenter O. Therefore, the Z-coordinates are zero for point N, G and P. Point N is obtained by projecting of mechanical leaf position E onto the scoring plane. Point G is obtained by deriving the tangent line of circular arc leaf end from source S. Point P is obtained by rule of half-value layer.

Equations for the LPO, RFO and PNO derivation are presented,

[1] $$\{\begin{array}{l} \mathrm{LPO}=x_{\text{G}}-x_{\text{N}}\\ \mathrm{RFO}=x_{\text{P}}-x_{\text{G}}\\ \mathrm{PNO}=x_{\text{P}}-x_{\text{N}} \end{array}$$

Algorithms for calculation the X-coordinates of leaf positions are illustrated as follows. Consider a specific nominal leaf position, which is designated as point N in Figure 1, Point E is obtained by back-projecting leaf end point N onto the collimator middle plane, that is

[2] $$x_{\text{E}}=x_{\text{N}}\times \frac{\text{SCD}}{\text{SAD}}$$

where SCD is used to stand for source to collimator distance, while SAD stands for source to axis distance. The circular arc center C is shifted to the positive side of point E with a length of radius R, that is,

[3] $$x_{\text{C}}=x_{\text{E}}+R$$

Denote the distance between source S and arc centre C as D, which is used as a reference,

[4] $$D=\sqrt{{\left(x_{\text{S}}-x_{\text{C}}\right)}^{2}{\left(z_{\text{S}}-z_{\text{C}}\right)}^{2}}$$

Firstly, point G is obtained by deriving the tangent line of circular arc leaf end from source S. The relationship between point G and point T is,

[5] $$x_{G}=x_{T}\times \frac{\text{SAD}}{\text{SAD}-z_{\text{T}}}$$

Thus, the prerequisite for point G derivation is to obtain point T. The X-coordinate of point T should satisfy the condition of *x*_T_ < *x*_C_. Point T can be obtained by the following equations,

[6] $$\{\begin{array}{l} {\left(x_{\text{T}}-x_{\text{C}}\right)}^{2}+{\left(z_{\text{T}}-z_{\text{C}}\right)}^{2}=R^{2}\\ \left(z_{\text{T}}-z_{\text{S}}\right)\left(z_{\text{T}}-z_{\text{C}}\right)+\left(x_{\text{T}}-x_{\text{S}}\right)\left(x_{\text{T}}-x_{\text{C}}\right)=0 \end{array}$$

In case that the tangent point falls out of circular arc or S falls within circle, denote the tangent point as the intersection point of circular arc with proximal or distal leaf side. The intersection points are depicted as point U and point V, respectively. Algorithm is illustrated as follows, for leaf height of *lh*,

[7] $$\{\begin{array}{l} x_{\text{T}},\;\;\text{if}\;z_{\text{T}}\in \left[z_{\text{C}}-lh/2,z_{\text{C}}+lh/2\right]\\ x_{\text{C}}-\sqrt{R^{2}-{\left(lh/2\right)}^{2}},\;\;\text{otherwise} \end{array}$$

[8] $$z_{\text{T}}=\{\begin{array}{l} z_{\text{C}}+lh/2,\;\text{if}\;D\leq R\\ z_{\text{C}}+lh/2,\;\text{if}\;D>R\;\;\text{and}\;\;z_{\text{T}}>z_{\text{C}}+lh/2\\ z_{\text{C}}-lh/2,\;\text{if}\;D>R\;\;\text{and}\;\;z_{\text{T}}>z_{\text{C}}-lh/2\\ z_{\text{T}},\;\text{if}\;D>R\;\;\text{and}\;\;z_{\text{T}}\in \left[z_{\text{C}}-lh/2,z_{\text{C}}+lh/2\right] \end{array}$$

Secondly, point P is obtained by the half-value layer rule. Draw a secant line from source S to the point P, the secant point A and B fall on the left side of C, that is, *x*_A_ < *x*_C_, *x*_B_ < *x*_C._ Find the equation of secant line satisfying the condition that path length AB equals half-value layer *L*,

[9] $$\{\begin{array}{l} L=-\text{ln}\left(0.5\right)/\mu \\ {\left(z_{\text{A}}-z_{B}\right)}^{2}+{\left(x_{\text{A}}-x_{\text{B}}\right)}^{2}=L^{2} \end{array}$$

where path length is calculated according to the inverse exponential power law and m denotes the attenuation coefficient of tungsten leaf. A, B and S are on the secant line. It is written in the following form,

[10] $$\left(z_{\text{A}}-z_{\text{S}}\right)\left(x_{\text{B}}-x_{\text{S}}\right)-\left(z_{\text{B}}-z_{\text{S}}\right)\left(x_{\text{A}}-x_{\text{S}}\right)=0$$

Suppose the A and B are both on the circular arc, that is,

[11] $$\{\begin{array}{l} {\left(x_{\text{A}}-x_{\text{C}}\right)}^{2}+{\left(z_{\text{A}}-z_{\text{C}}\right)}^{2}=R^{2}\\ {\left(x_{\text{B}}-x_{\text{C}}\right)}^{2}+{\left(z_{\text{B}}-z_{\text{C}}\right)}^{2}=R^{2} \end{array}$$

Combine [9], [10] and [11] into a system of nonlinear equations, solve it with iterative methods. In case that the resulting A or B is not on circular arc, conditional statements are performed as follows.

If the resulting *Z*_A_ > *Z*_C_ + *lh* /2 or *D* < *R*, it means point A should be on the proximal leaf side. Substitute equation [12] for [11] in the system of equations and solve it.

[12] $$\{\begin{array}{l} {\left(x_{\text{A}}-x_{\text{C}}\right)}^{2}+{\left(z_{\text{A}}-x_{\text{C}}\right)}^{2}=R^{2}\\ {\left(x_{\text{B}}-x_{\text{C}}\right)}^{2}+{\left(z_{\text{A}}-z_{\text{C}}\right)}^{2}=R^{2} \end{array}$$

Else, if *Z*_B_ < *Z*_C_ −*lh*/2, substitute equation [13] for [11] in the system of equations and solve it.

[13] $$\{\begin{array}{l} {\left(x_{\text{A}}-x_{\text{C}}\right)}^{2}+{\left(z_{\text{A}}-z_{\text{C}}\right)}^{2}=R^{2}\\ z_{\text{A}}=z_{\text{C}}-lh/2 \end{array}$$

Else, return with the solutions of equations.

### Monte Carlo simulation

Numerical simulation is performed based on Monte Carlo codes EGSnrc/BEAMnrc.^15^,^16^ Multileaf collimator component module of VARMLC is adopted. Leaf ends in the shape of circular arc are used, with radius values in a range of 4 cm to 25 cm. The geometry of Monte Carlo simulation is depicted as Figure 1. Multileaf collimator is comprised of 40 pairs of leaves. Symmetric field size of 10x10 is adopted, which remains constant for different leaf positions. It means that the central 10 pairs of leaves shift for field shaping, while the peripheral leaves stay still in close state. For central leaf pair, the gap between leading leaf and trailing leaf corresponds to the square field edge of 10 cm, measured along leaf travel direction on scoring plane. Diaphragms are extracted to the maximum position in order to avoid interference with radiation beams. Source sizes with a range of 0.5 to 3 mm full width at half maximum (FWHM) are adopted. Parameters for configuration are listed in Table 1.

### Data processing

**FIGURE 2.**
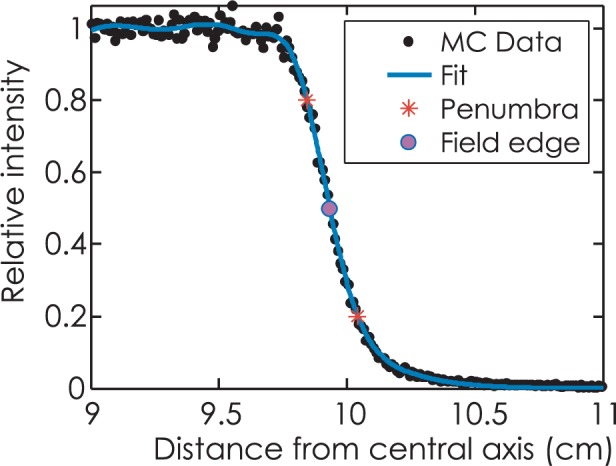
Data processing for leaf end radius of 10 cm with nominal leaf position at 10 cm and source size of 2 mm full width at half maximum. MC = Monte Carlo

Data processing software BEAMDP is utilized for deriving energy fluence versus position from the acquired phase space files grouped by source size and leaf end radius. Curve fitting is performed to obtain penumbra characteristics, including penumbra width and radiation field offset. Figure 2 shows a sample result for data processing.

Normalize the Monte Carlo data by scaling between 0 and 1. The penumbra width is referred to as the distance between relative intensity of 0.2 and 0.8, and the radiation field edge is referred to as the position with relative intensity of 0.5. Gaussian function is employed for curve fitting, with the following equation:

[14] $$f\left(x\right)=\sum_{i=1}^{n}a_{i}e^{-{\left(\left(x-b_{i}\right)/c_{i}\right)}^{2}}$$

Three-peak Gaussian curve fitting is used for the sample data, with coefficients listed in Table 2. Result shows that the goodness of fit is 0.0154 measured by Root Mean Squared Error. Penumbra width is 1.98 mm and radiation field edge is at 9.932 cm, with PNO offset of 0.68 mm.

## Results

### Penumbra width

**FIGURE 3A–D:**
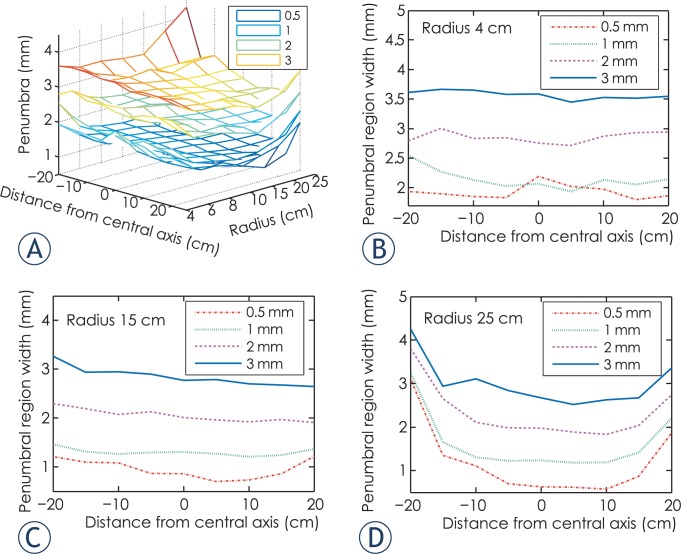
**(A).** 3D graph of penumbra width with source size of 0.5 to 3 mm full width at half maximum. **(B).** Penumbra width for leaf end of radius 4 cm. **(C).** Penumbra width for leaf end of radius 15 cm. **(D).** Penumbra width for leaf end of radius 25 cm.

The results of penumbra width are illustrated as Figure 3A. Note that penumbra width varies according to field location, leaf end radius and source size. The minimum and maximum penumbra width are obtained with source sizes of 0.5 mm and 3 mm FWHM, respectively. Figures 3B, 3C and 3D show penumbra width for leaf end radius of 4, 15, 25 cm, respectively. Observe that penumbra width is a function of distance from central axis. The overall trend is that penumbra width with positive distance from central axis is smaller than its negative counterpart.

**FIGURE 4.**
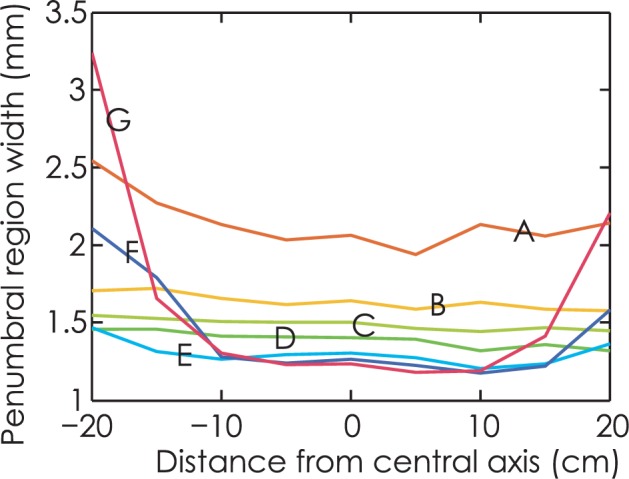
Penumbra width for source size of 1mm full width at half maximum. Curve A to G denote radius of 4, 6, 8, 10, 15, 20, 25 cm.

Figure 4 shows the penumbra width with for source size of 1 mm FWHM. Curve E with leaf end radius of 15 cm demonstrates the minimum sum of penumbra width, which is the optimum in terms of penumbra characteristics. Observe that for large radius leaf end and nominal leaf position far from central axis, penumbra width curve are U-shaped, as shown by curve F and curve G.

### Radiation field offset

**FIGURE 5A–D:**
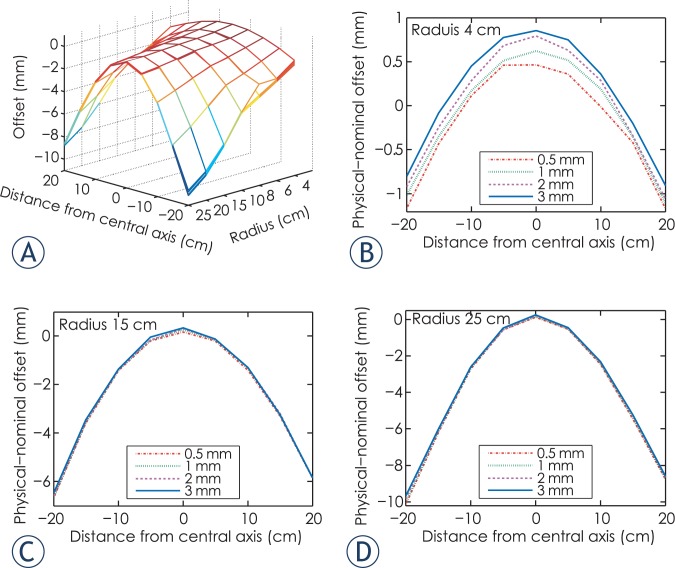
**(A).** 3D graph of physical-nominal offset with source size of 0.5, 1, 2, 3 mm full width at half maximum. **(B).** physical-nominal offset (PNO) for radius of 4 cm. **(C).** PNO for radius of 15 cm. **(D).** PNO for radius of 25 cm. Source size ranges from 0.5 to 3 mm.

Results of physical-nominal offset are depicted in Figure 5A. It is noted that source size has little impact on physical-nominal offset and the surfaces of PNO with source size of 0.5, 1, 2, 3 mm FWHM coincide with each other. Physical-nominal offset maximum of −10.1 mm is obtained at radius of 25 cm with nominal leaf position at −20 cm. Figures 5B, 5C and 5D show PNO for source size of 0.5 to 3 mm with leaf end radius of 4 cm, 15 cm and 25 cm, respectively. Observe that the maximum discrepancy of PNO curves occurs at central axis for radius of 4 cm, which is 0.4 mm.

**FIGURE 6.**
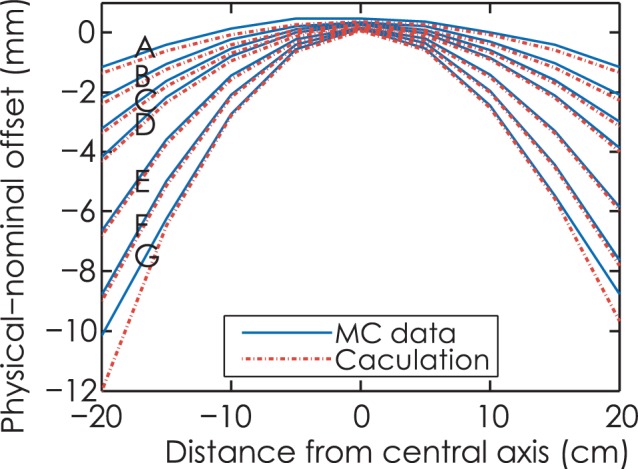
Physical-nominal offset for 1mm full width at half maximum source. Curve pair A to G are referred to as circular arc leaf ends with radius of 4, 6, 8, 10, 15, 20, 25 cm, for Monte Carlo (MC) simulation (solid line) and analytical method (dashed line).

Figure 6 shows the physical-nominal offset of Monte Carlo simulation and calculation using analytical method for source size of 1 mm FWHM. Note that PNO curve for small radius tends to be flat, and leaf end with large radius follows a quasi-quadratic function between PNO and leaf projected position. Although overall trends of calculation curves are in good agreements with Monte Carlo data, the results of Monte Carlo data are slightly larger compared with analytical values, with maximum discrepancy of 2 mm found for curve pair G at the point with a distance of −20 cm from central axis.

**FIGURE 7.**
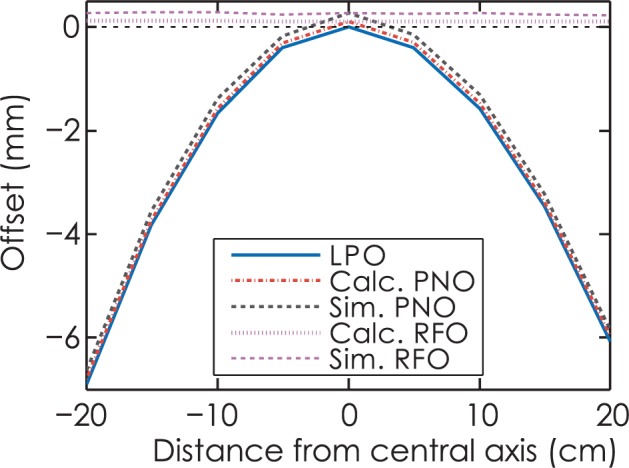
Comparison of leaf position offset (LPO), physical-nominal offset (PNO) and radiation field offset (RFO) for leaf end of radius 15 cm with source size of 1 mm. Calc. = calculation; Sim. = simulation

As illustrated in Figure 7, the analytical results agree well with Monte Carlo simulation for leaf end of radius 15 cm with source size of 1 mm. The curves of calculation PNO and RFO are shifted upwards with a constant gap of 0.16 mm, compared with simulation PNO and RFO. Note that the RFO curve is almost parallel with axis, which implies that a constant RFO value could be assigned and physical-nominal offset could be quickly deduced from leaf position offset. The RFO values derived from analytical method and numerical simulation are 0.10 and 0.26 mm, respectively.

**FIGURE 8.**
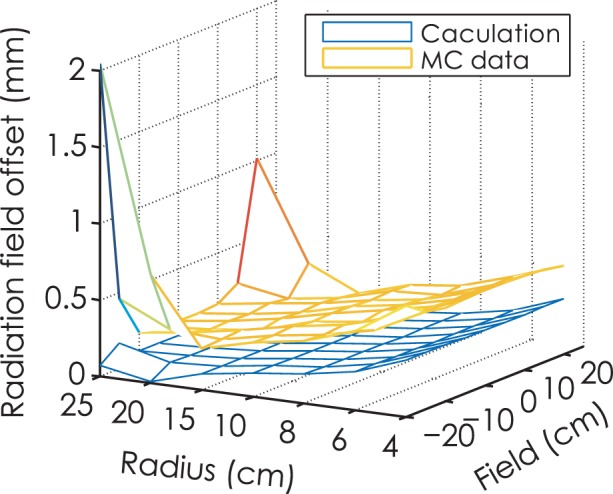
Comparison of radiation field offset (RFO) for Monte Carlo simulation and analytical method with source size of 1 mm full width at half maximum. MC = Monte Carlo

Figure 8 shows the radiation offset results using Monte Carlo simulation and analytical method. It is noted that the RFO values of Monte Carlo data are slightly larger than the calculation data. As for leaf end with radius of 25 cm, the curve of radiation field offset is U-shaped with maximum RFO of 2 mm.

## Discussion

The results demonstrate that algorithms for leaf position calculation and radiation field offset correction serve well the purpose of investigating rounded leaf end effect on penumbra characteristics. Compared with previous works^10^–^14^, the algorithms we proposed are advantageous particularly in dealing with large radius leaf end. It is shown that penumbra width of multileaf collimator is a function of radiation source size, geometry of treatment head, leaf end shape and projected leaf position on scoring plane. Optimal radius of leaf end shape could be found by examining the penumbra width curves of Monte Carlo simulation. The results also reveal that radiation source size has a negligible impact on radiation field offset, while for penumbra width, source size counts.

In our study, virtual source model (VSM) is applied, which has been intensively reported by previous studies.^16^ The VSM technique for dose calculation is computational efficient and able to simulate the same dose profile without explicitly taking into consideration the realistic treatment head geometry. Virtual source in our study is Gaussian shaped. However, for realistic treatment head, source energy distribution could more complex than single Gaussian source. In order to accommodate realistic system properties, firstly, virtual source modelling should be conducted to identifying focal source and extra-focal source energy distribution. Secondly, Monte Carlo simulation would be performed according to results of VSM. The geometry of treatment head for numerical simulation is simplified so that the impact of treatment head components, such as primary collimator and flattening filter, on extra-focal radiation, is minimized. However, it is quick to implement by modifying the component properties in Monte Carlo simulation codes.

Monoenergetic photon source is designated as Gaussian shaped with full width at half maximum in a range of 0.5 mm to 3 mm, which is in accordance with the dosimetric results.^17^ Average source energy of 1.5 MeV is adopted corresponding to 6MeV medical linear accelerator. However, energy spectrum and angular distribution of radiation beams would be implemented in the future works, which is not a trivial task.

For leaf end with large radius, the U-shaped curves appear both for penumbra width and radiation field offset, which are not preferred for clinical application. This observation is probably related with the fact that radiation beams penetrated through the proximal and distal leaf sides.

Although the fluence energy distribution has been intensively studied, the round leaf end effect on penumbra characteristics in phantom or *in vivo* has not been explored, which could be realized by dose calculation algorithms in future works. Scatter effect on dose profile could be calculated using kernel-based convolution and superposition algorithms.

It is noted that the results of RFO correction algorithm are generally in good agreement with numerical simulation. However, the values obtained using analytical method are slightly smaller compared with the corresponding Monte Carlo results, which means that analytical method may underestimates radiation field offset. In order to better predict the radiation field offset, analytical RFO can be placed to match numerical RFO by moving in the direction of from irradiation area to shielded area. This observation implies that analytical method should be applied with care. The error between analytical and numerical methods is probably related the empirical rule of half-value layer or “geometric optics” formulae. A simple physical explanation for the underestimation is given as follows.

Denote P as the physical leaf position obtained by “geometric optics” formulae, which means the path length AB equals half value layer. Denote P_50_ as the radiation field edge, or physical leaf position, obtained by Monte Carlo simulation. Consequently, the question being proposed could be rephrased as “why is *x*_P_ < *x*_P_50__?”.

**FIGURE 9.**
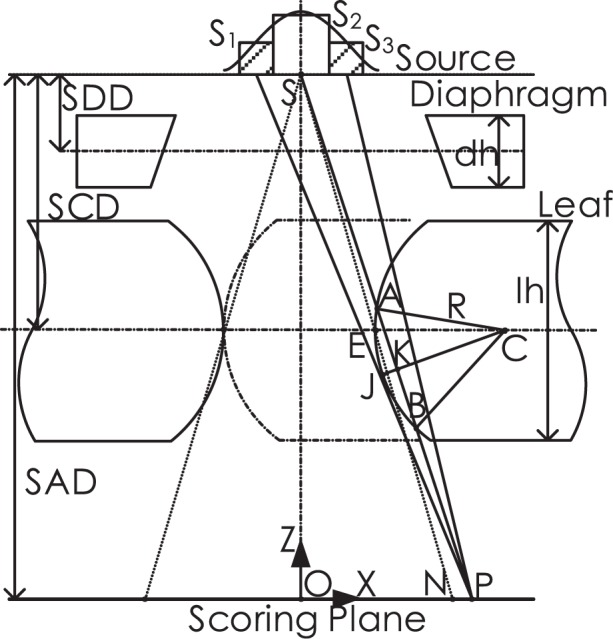
Geometry of treatment head for simple physical explanation of why the rule of half value layer systematically underestimates the physical-nominal offset. SAD = source to axis distance; SCD = source to collimator distance; SDD = source to diaphragm distance

Firstly, as illustrated in Figure 9, draw a line CJ from circle arc centre C that is perpendicular with path length AB, with intersection point of K. Denote the length of JK as *H*. It is obvious that *H* is monotonically decreasing for R > 0. For clinical application, leaf end radius is commonly larger than half of leaf width. The maximum of *H* is written,

[15] $$H=R-\left|\bar{\text{CK}}\right|=R-\sqrt{R^{2}-{\left(\left|\bar{\text{AB}}\right|/2\right)}^{2}}<8/2-\sqrt{{\left(8/2\right)}^{2}-{\left(-\text{ln}(0.5)/0.96/2\right)}^{2}}=0.016\;\text{cm}$$

Normally, source energy distribution for treatment modality is with FWHM ranging from 1 to 3 mm. Note that the H is small compared with source size.

Secondly, suppose that source energy distribution is approximately symmetric about central axis. Divide source energy into three parts, the left part S_1_, the middle part S_2_ and the right part S_3_ with respect to the central axis. Denote that total source energy as 1, it is written that,

[16] $$S_{1}+S_{2}+S_{3}=1$$

[17] $$S_{1}=S_{3}$$

The attenuation weight for the beams from source part S_1_, S_2_ and S_3_ to the point P are defined as *w*_1_, *w*_2_ and *w*_3_, respectively. The rule of half value layer tells that *w*_2_ = 0.5. On account that H is small, beams irradiate from the left part of source are supposed to reach P without attenuation, that is *w*_1_ = 1, while for the right part, beams penetrate through leaf entity to reach P with path length larger than half value layer, that is, 0 < *w*_3_ < 0.5. Consequently, the radiation intensity *E*_P_ of point P is written as follows,

(18) $$E_{\text{P}}=w_{1}S_{1}+w_{2}S_{2}+w_{3}S_{3}>S_{1}+0.5\cdot S_{2}=0.5\cdot \left(S_{1}+S_{2}+S_{3}\right)=0.5$$

Therefore, it is implied that the physical edge of radiation field P_50_ should be on the right side of P, that is, *x*_P_ < *x*_P_50__. This is a simple physical explanation why “geometric optics” formulae systematically underestimate the physical-nominal offset. Since the segmentation of source energy is coarse, further study is suggested with Ray Tracing algorithm, which is implemented by computation of the weighed beam integral based on the law of exponential attenuation. It is suggested that modifications for analytical RFO correction should be performed in order to fit in well with treatment modalities. Path length larger than the half-value layer would be beneficial.

The rounded leaf end design of multileaf collimators leads to partial transmission of radiation beams, which have a significant impact on dose delivery accuracy of IMRT, SBRT and VMAT. Based on Monte Carlo simulation for SBRT multileaf collimator, Asnaashari *et al.*^5^ have revealed that dosimetric penumbra is influenced by source energy, beam collimators and field size. This observation is in good agreement with our study. It is suggested that dosimetric characteristics of multileaf collimator should be calibrated and comprehensive routine quality assurance should be performed before they are implemented for IMRT applications.^3^ Further study is needed both for theoretical investigation and dosimetric measurement of rounded leaf end effect.

In our study, penumbra width and radiation field offset of single leaf are intensively studied. In contrast, Szpala *et al.*^11^ investigated the value of dosimetric leaf gap (DLG) for leaf pairs in treatment planning. It was demonstrate that the DLG depends on the size of mulileaf collimator slit. Such effect is probably caused by scatter variation from the opposite leaf with different slit widths. Furthermore, they proposed a method by expanding the DLG parameter from a single value to a function of distance from the nominal leaf position and displacement of the opposite leaf. However, efforts should made to improve dose calculation accuracy in VMAT treatment planning, not merely by adjusting single parameter, such as leaf transmission or DLG. Better modeling rounded leaf end effect is of significance for future works.

## Conclusions

In summary, the algorithms we proposed for leaf position calculation and radiation field offset correction are effective for leaf end with large radius. Results of Monte Carlo simulation show that source size influences penumbra width, while for radiation field offset, the source size impact is negligible. Penumbra width performance could be improved by carefully choosing the radius of circular arc leaf end. In this study, the leaf positions, including mechanical leaf position, nominal leaf position, geometric leaf position and physical leaf position are classified and rigorously deduced. Correction of leaf position offset, radiation field offset and physical-nominal offset are realized based on analytical method. In general, results of analytical method agree well with numerical simulation. However, a slight gap exists between analytical radiation field offset and numerical radiation field offset, which implies that modification should be introduced when applying the empirical rule of half-value layer. For better treatment planning, the rounded leaf end effect on penumbra characteristics should be taken with care in order to achieve dose delivery accuracy.

## Figures and Tables

**TABLE 1.** Parameters for Monte Carlo simulation

**Parameter**	**Value**	**Unit**
Source to axis distance	100	cm
Source to collimator distance	46	cm
Source to diaphragm distance	33.9	cm
Diaphragm height	7.8	cm
Leaf height	8	cm
Leaf pairs	40	-
Leaf density	19.3	g/cm^3^
Attenuation coefficient m	0.96	cm^−1^
Maximum field size	40	cm
Photon source average energy	1.5	MeV
Source divergence angle	15.8°	-
Recording histories	10^9^	-

**TABLE 2.** Three-peak Gaussian curve fitting coefficients

**Coefficient**	**Value**	**Coefficient**	**Value**	**Coefficient**	**Value**
a_1_	0.406	a_2_	0.397	a_3_	0.996
b_1_	9.813	b_2_	9.586	b_3_	9.054
c_1_	0.169	c_2_	0.269	c_3_	0.668
